# Characterization of Ty21a immunostimulatory effects in the mouse bladder

**DOI:** 10.3389/fimmu.2025.1629462

**Published:** 2025-11-26

**Authors:** Lenka Polak, Rim Hojeij, Valerie Cesson, Jacques-Antoine Haefliger, Thierry Roger, Ilaria Lucca, Laurent Derré, Denise Nardelli-Haefliger, Sonia Domingos-Pereira

**Affiliations:** 1 Urology Research Unit, Department of Urology, Lausanne University Hospital and University of Lausanne, Lausanne, Switzerland; 2 Department of Medicine, Lausanne University Hospital and University of Lausanne, Lausanne, Switzerland; 3 Infectious Diseases Service, Department of Medicine, Lausanne University Hospital and University of Lausanne, Epalinges, Switzerland

**Keywords:** Ty21a salmonella formulation, bladder cancer, intravesical immunotherapy, T-cell infiltration, vessel permeability, chemokine, TLR

## Abstract

Intravesical treatment with *Salmonella enterica* Ty21a, an oral typhoid-fever vaccine, has shown therapeutic potential against bladder tumors mainly through local immune-cell recruitment, particularly CD8^+^ T-cells. However, the mechanisms underlying its efficacy and the impact of bacterial formulation remain unclear. Here, we show that increased immune-cell infiltration was neither associated with modification in blood vessel density nor the generation of high endothelial venules, but rather with a transient increase in local vessel permeability, requiring live bacteria. Giving prior evidence that freshly harvested bacteria (Ty21a^FR^) were more efficient than lyophilized bacteria (Ty21a^LYO^), we tested both formulations intravesically in mice. Although, both similarly increased vascular permeability, Ty21a^FR^ induced significantly greater immune-cell recruitment locally and more effective tumor regression in the orthotopic MB49 bladder cancer model. Chemokine analysis showed higher levels of C5a, CXCL2 and CXCL5 in Ty21a^FR^-treated bladders, however their receptors (C5aR, CXCR2) were barely detected on infiltrating T cells, precluding their direct involvement in T-cell recruitment. Instead, Ty21a^FR^ increased C5aR^+^ and C5aR^-^CD11b^high^ myeloid cells, suggesting their indirect influence on T-cell recruitment. We hypothesized that LPS, a TLR4 agonist, from *Salmonella*, might be involved. Indeed, CD8^+^ T-cell infiltration following Ty21a^FR^ was significantly decreased in TLR4- and MyD88-KO mice. In contrast, myeloid-cell recruitment was only reduced in MyD88-KO mice, suggesting the involvement of TLR4-independent pathways in that process. This study is the first to identify Ty21a formulation-driven immunostimulatory differences in bladder cancer. Altogether, our data provide new insights into Ty21a’s immunostimulatory mechanisms and highlight the importance of bacterial formulation for optimizing bladder cancer treatment.

## 1 Introduction

Bladder cancer is a common urologic malignancy that is in part caused by smoking habits and exposure to industrial chemicals and shows an increased incidence in the elderly population (1–3). Although, seventy percent of bladder cancers are diagnosed as non-muscle-invasive and are treated by transurethral resection of the bladder tumor lesion, they have a high propensity to recur and/or progress to muscle invasive cancer. Non-muscle invasive bladder cancer (NMIBC) patients with high risk for progression receive, as a gold standard treatment, intravesical instillations with live Bacille Calmette Guérin (BCG) bacterial vaccine, effectively reducing recurrence/progression (4). Although BCG therapy is one of the most successful immunotherapies in use, 5-year recurrence free survival is only 40-60% (5, 6), treatment causes significant side-effects (7) and is subject to frequent shortage (8), emphasizing the need for alternative or complementary therapies (9). Another bacterial vaccine, the highly attenuated *Salmonella enterica* serovar Typhi strain Ty21a (10) included in Vivotif^®^, a commercial oral vaccine against typhoid fever, has recently shown potential for intravesical application. Indeed, intravesical Ty21a safely and effectively induces bladder tumor regression in the orthotopic MB49 bladder tumor model (11, 12). A Phase I trial (NCT03421236) in NMIBC patients with low/intermediary risk of recurrence/progression confirmed the favorable safety profile of Ty21a (13) as well as the generation of robust, potentially anti-tumor immune responses (14).

In combination therapies, a common strategy to enhance vaccine-specific T-cell recruitment to the tumor involves administration of immunostimulants or danger signals such as chemokines (15) or Toll-like receptor (TLR) agonists (16) at the mucosal or tumor site following systemic vaccination. We demonstrated that such strategies may be effective in mouse orthotopic models including cervical cancer, where intravaginal administrations of TLR agonist or *Salmonella* after subcutaneous (s.c.) HPVE7 vaccination (17, 18) induced local T-cell recruitment, and bladder cancer, where intravesical instillation of CpG, PIC, BCG or *Salmonella* following s.c. HPVE7 vaccination (used as a model tumor vaccine) yielded similarly beneficial effects (19). In addition, as a proof-of-principle, we demonstrated the efficacy of such a strategy in NMIBC patients treated with intravesical BCG in combination with intramuscular MAGE-A3 cancer vaccine (20). Recruitment of immune cells at mucosal sites, including the bladder, rely not only on homing molecules/integrins expressed on circulating immune cells and their counterpart adressins on blood vessels (21), but also on chemokine cross-talk (22). Angiogenesis leads to the formation of new tumor blood vessels, which are often leaky, disorganized and poorly efficient. This process plays a key role in tumor development and metastasis (23). Moreover, it has been suggested that normalizing tumor vasculature may enhance the delivery therapeutic drug to the tumor and reduce metastatic progression (24–26). Vascular remodeling upon inflammation, such as generation of new vessels and high endothelial venules (HEV) (27), or increased vessel permeability (28), are also well-known mechanisms that facilitate immune cell infiltration (29). We therefore investigated the influence of intravesical Ty21a on these processes.

In previous reports evaluating the therapeutic potential of local administration of Ty21a against cervical or bladder cancer, we used freshly prepared Ty21a bacteria (Ty21a^FR^) (17–19). However, more recently, the content of the commercially available Vivotif^®^ capsule, which consists in a lyophilized form of Ty21a (Ty21a^LYO^), has been employed towards its approval for intravesical treatment of bladder cancer patient in clinical trials (11–14). During the development of oral vaccination against typhoid fever, data suggested that Ty21a^FR^ was more efficient than Ty21a^LYO^ (30, 31), although the latter was eventually developed for commercial use in mass oral vaccination. To determine which formulations of Ty21a may be more efficient for the treatment of bladder cancer, here we compared Ty21a^FR^ vs Ty21a^LYO^ administrated via intravesical route in mice, evaluating their effects on vessel permeability, immune cell infiltration, chemokine induction and bladder tumor regression.

## 2 Materials and methods

### 2.1 Mice and tumor cell line

Seven to ten-week-old female C57BL/6 wild-type mice (Envigo) were used and all experiments were performed in accordance with Swiss law and with approval of the Cantonal Veterinary Office of Canton de Vaud, Switzerland (license VD-1046). The MB49 cell-line (kindly provided by Prof. A. Loskog, Uppsala University, Sweden) is derived from a carcinogen induced urothelial carcinoma in male C57BL/6 mice (32). Luciferase-expressing (MB49-luc) cells were generated by transfection with lentiviral vector encoding for firefly luciferase (kindly provided by Prof. D. Trono, EPFL, Lausanne, Switzerland). Female MyD88 KO (33) and TLR4 KO (34) mice were housed under specific pathogen-free conditions in the animal facility of Epalinges, Switzerland (license VD-H04).

### 2.2 Intravesical instillation

Deeply anesthetized mice received at the indicated time point a single intravesical instillation of PBS (50µl) or bacterial solutions (3x10^8^ colony forming unit (CFU)) (11), using an Introcan 24G/3/4 (Braun, Melsungen, Germany) catheter inserted after lubrication with K-Y ^®^ gel. S. *enterica* serovar Typhi Ty21a lyophilized bacteria (Ty21a^LYO^) in the format contained within the enteric-coated capsule used for oral immunization (Vivotif^®^ (Bavarian Nordic Berna, Thörishaus, Switzerland) including >2×10^9^ CFU Ty21a, sucrose (7.9–44 mg), lactose (max 176.4mg), ascorbic acid (E300), acid casein hydrolysate, magnesium stearate (E470)) were used within 1 h after reconstitution and dilution in PBS. For Ty21a^FR^, Ty21a bacteria were grown in LB (Luria-Bertani) media at 37 °C to OD_600_ = 0.6, concentrated by centrifugation and resuspended in PBS at the desired concentration for fresh administration (10, 35). Bacterial concentrations of Ty21a^FR^ were confirmed by serial dilution and plating on LB agar plates, followed by colony enumeration after overnight incubation at 37°C.

### 2.3 Immunohistochemistry

Mice were sacrificed by CO_2_ inhalation. Bladder was intravesically instilled with an optimal cutting temperature (OCT) compound before mechanical dissociation and freezing into liquid nitrogen. Ten µm-thick transversal sections were stored at -80°C. For immunological staining, the bladder sections were quickly fixed 5 minutes in ice-acetone and dried for 5 minutes. The sections were then washed with PBS three times and each individual section was circled using a Dako-Pen. The cryosections of the bladder were incubated in blocking buffer (PBS, 5% BSA, 2.5% FCS) for 45min at room temperature (RT) in a humidity chamber and then incubated overnight at 4°C with one of the primary antibodies against Pnad (Meca79, Biolegend), CD31 (MEC 133, BD Pharmigen) or CD8 (10-0081-82, Bioscience), which were diluted in 1% BSA PBS solution. After 3-times washing with PBS under slight agitation, the bladder sections were incubated for 45 minutes at RT in a humidity chamber with corresponding Alexa-Fluor-488-conjugated secondary antibodies: anti-rat IgG (A11006) or anti-rat IgG (A21208, all from Life technologies). The sections were further washed three times with PBS and covered with PBS containing 50% of glycerol and DAPI diluted to 1:10 (Duolink; 82040 0005). After mounting, the slides were observed by fluorescence microscopy (Leica DMI3000B, DF345FX) with the respective software (Leica Application Suite). The whole tissue area of each bladder section was examined (8–10 microscopic fields) and the total count of CD31+ vessels or CD8 T cell numbers were shown in the graphs (i.e. numbers/bladder section). Pictures were analyzed by ImageJ.

### 2.4 Hemoglobin content

Bladders were frozen and powdered in liquid nitrogen. The weighed bladder powder was homogenized in 100µl of 0.1% Brij L23 solution and incubated 2 minutes at RT. After centrifugation (5 minutes, 15’000 g, RT), 50µl of the supernatant was transferred to 450 µl of Drabkin’s reagent solution (Sigma-Aldrich, D5941-6VL). The absorbance was measured by spectrometry at 540 nm. The concentration of hemoglobin was then determined from the standard curve of cyan-methemoglobin.

*In vivo permeability assay.* Mice were injected with 200 µl of 0.5% of Evans Blue solution (Sigma, E2129) in lateral tail vein (36). After 30 minutes, the mice were sacrificed through cervical dislocation. Different tissues such as the bladder, the lung and the liver were collected, weighed and incubated in 500 µl of formamide (Sigma, F9037) during 24h in water bath at 55 °C. After centrifugation (5 minutes, 15’000 g, RT), the absorbance was measured at 610 nm by spectrophotometry and Evans Blue content calculated with a standard curve.

### 2.5 Immunostaining and flow cytometry analysis

Mice were sacrificed by CO_2_ inhalation to collect the bladders. Single-cell suspensions were obtained by mincing in DL-dithiothreitol (Sigma, D9779) and by subsequent digestion with 1 mg/ml collagenase/dispase (Roche, 11097113001) and 0.1 mg/ml DNAse I (Sigma-Aldrich, D5025) with 20% fetal calf serum (Gibco, 10270). The recovered cells were stained and analyzed by flow cytometry. The following monoclonal anti-mouse antibodies were used: anti-CD3-PerCP/Cy5.5 (17A2) (Biolegend, 100218), anti-CD4 (RM4-5) FITC or eF450 (eBioscience, 11-0042–81 or 48-0041-82), anti-CD8 (53-6.7) APC (eBioscience, 17-0081-82) or PE-TXRD (Southern Biotech, 1550-10), anti-CD11b (M1/70) FITC (eBioscience, 101206) or APC (Biolegend, 101212), anti-CD88(C5aR)-PE-Cy7 (20/70) (Biolegend, 135809), anti-CXCR2-PE (242216) (R&D, FAB2164P). Uty-specific cells were stained using the H-2Db restricted dextramer Uty_246-254_-PE (Immudex). Dead cells were excluded by a live/dead fixable kit: aqua dead cell stain kit (L34957, Invitrogen, Thermo Fisher Scientific). Cell acquisition and analysis were performed using Gallios Flow Cytometer (Beckman Coulter, Nyon, Switzerland) and FlowJo software (Tree Star, Ashland, OR), respectively.

### 2.6 Vaccination and IFN-*γ* ELISPOT assay

The minor histocompatibility male antigen HY (Uty) is expressed by the MB49 bladder tumor cell line. The H-2Db-restricted epitope Uty_246–254_ peptide (37) (WMHHNMMDLI) was synthesized by the Peptide and Tetramer Core Facility of the department of oncology (Lausanne University Hospital and University of Lausanne, Switzerland). Mice were immunized s.c. with 50 μg of Uty_256–254_ adjuvanted with 0.4 μg heat labile enterotoxin (kindly provided by Berna-Biotech, Bern, Switzerland) and 10μg of CpG (#1826, 5’-TCCATGACGTTCCTGACGTT-3’, Coley Pharmaceutical Group). IFN-γ ELISPOT assays were performed as previously described (38) using Multi-screen-HA 96-well plates (MAHAS4510, Millipore) anti–IFN- γ mono-clonal antibody (R4-6A2, Beckton Dickinson PharMingen), biotinylated anti–IFN- γ monoclonal antibody (XMG1.2, Beckton Dickinson PharMingen), and Streptavidin-AP (Roche). In brief, 3x10^4^ bone-marrow-derived DCs (BMDCs)/well, used as antigen-presenting cells, were incubated for 1h in duplicate with 1mg/mL of Uty_256–254_ peptide or medium alone (control wells) before addition of 10^5^ bladder cells and incubation for 16–24h. Uty-specific responses were defined as the number of IFN- γ spots/10^5^ cells in the Uty-stimulated wells minus the number of IFN- γ spots/10^5^ cells in the control wells (<3 spots/well). BMDCs were generated from bone marrow cells in the presence of 150 U ml^−1^ of recombinant mouse granulocyte–macrophage colony-stimulating factor (R&D Systems, Abingdon, UK) as previously described (39).

### 2.7 MB49-orthotopic tumor model

Bladder tumors were established in deeply anesthetized mice that were uretherally catheterized using Introcan 24Gx3/4 catheters (Braun, Melsungen, Germany) as previously described (12). A 15 minutes pre-treatment with 100 μl 22% ethanol was performed before instillation of 500’000 MB49-luc cells in 50μl of Hank’s balanced salt solution (HBSS) (Gibco, 14025092). MB49-luc tumor growth was monitored by bioluminescence 15 minutes after intraperitoneal (i.p.) injection of D-luciferin (Promega, L8220, 150 μg/g of body weight) in the Xenogen imaging system (Xenogen/IVIS Caliper Life Science, kindly provided by cellular imaging facility, CIF/UNIL, Lausanne, Switzerland). All mice will develop bladder tumors and monitoring of MB49-luc tumors establishment and growth can be efficiently assessed during the first 3 weeks. Uncontrolled loss of luminescence of the growing tumors can then often appear (40), requiring additional monitoring by palpation, hematuria and overall health status of the mice, that were euthanized when they reached human endpoints.

### 2.8 Chemokine array

Bladders were recovered 24h after intravesical immunostimulation with 3x10^8^ CFU Ty21a^FR^ or Ty21a^LYO^ and homogenized in 500-1000 μL PBS with protease inhibitors (10 μg/mL Aprotinin from bovine lung, 10 μg/mL Leupeptin hemisulfate salt, and 10 μg/mL Pepstatin A; all from Sigma-Aldrich). TritonX-100 (final concentration 1%, Sigma-Aldrich) was added after homogenization, and after two freeze-thaw cycle’s samples were centrifuged at 10’000 g for 5 minutes to remove debris. Protein concentration was assessed using BCA protein assay (Thermo scientific). Chemokines were detected using the Proteome profiler array: mouse chemokine array kit (R&D Systems), according to the manufacturer instructions. Briefly, 150 μg of protein (pooled from three bladders, 50 μg each) of each condition were used for the assay. Detection of chemokine levels was performed using ImageJ software (NIH) and expressed as mean pixel density. Increased chemokine levels between Ty21a^FR^ and Ty21a^LYO^ were considered significant when ≥ to the 99% confidence interval of the mean fold-increases (i.e. ≥ 1.5 fold).

### 2.9 Statistics

Statistical analyses were performed using GraphPadPrism 10 for Windows (GraphPad software). Multiple comparisons were performed using one-way ANOVA and Tukey’s or Sidak post-test or log-rank test as indicated in the figure legends.

## 3 Results

### 3.1 Intravesical Ty21a increases blood vessel permeability, but not the generation of new vessels or HEV

**Figure 1:**
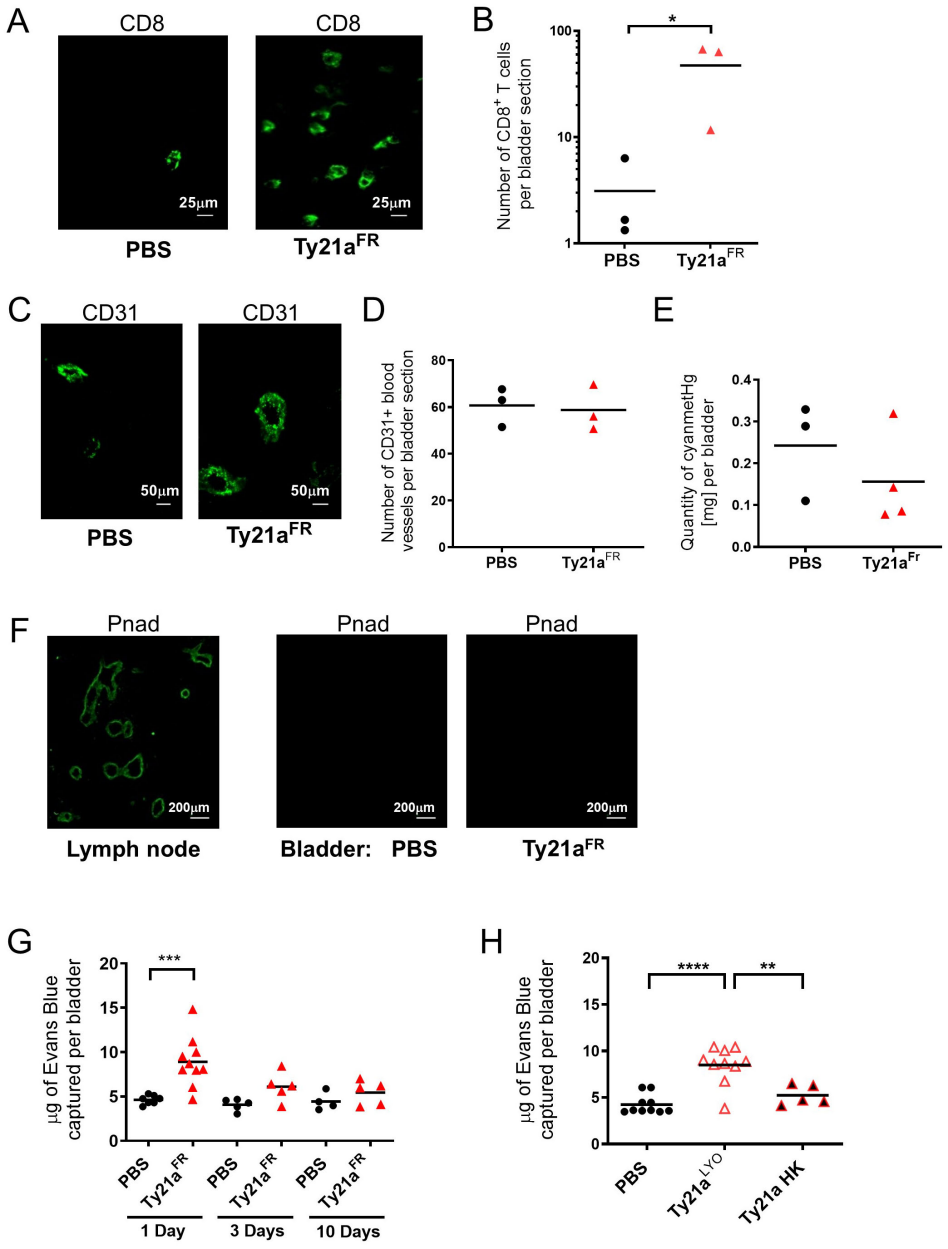
Ty21a instillation increases bladder blood vessel permeability. Naïve mice intravesically instilled once with PBS or $3\times 10^{8}$ CFU of Ty21a^FR^, were sacrificed 72h later and analyzed by immunohistochemistry **(A–D, F)** or for Hemoglobin content with a Drabkin assay **(E)**. **(A)** Representative CD8 staining of T cells in the bladder (PBS and Ty21a^FR^). Scale bar=25µm. **(B)** Mean numbers of CD8^+^ T cells per bladder section on three successive sections (n=3 mice/group). **(C)** Representative CD31 staining of bladder, scale bar=50µm. **(D)** Mean numbers of blood vessels per bladder section on three successive sections (n=3 mice/group). **(E)** Hemoglobin (cyanmetHg) content in mg/bladder (n=3–4 mice/group). **(F)** Representative Protein N-Terminal Asparagine Amidohydrolase (Pnad) stainings of high endothelial venule (HEV) in a lymph node section (left panel: positive control) or in the bladder (right panels, PBS and Ty21a^FR^), scale bar=200µm. **(G)** Evans Blue recovery (µg per bladder) in individual mice was examined after sacrifice at the indicated time points after intravesical instillations of PBS or $3\times 10^{8}$ CFU of Ty21a^FR^ (1 Day: n= 7–10 mice/group, 3 Days: n= 5 mice/group and 10 Days: n = 4–5 mice/group) **(H)** Evans Blue recovery (µg per bladder) 24h after intravesical instillation of PBS (n=10 mice/group), $3\times 10^{8\;}$ CFU Ty21a^LYO^ (n=10 mice/group) or Ty21a heat-killed (HK) (n=5 mice/group). Groups were compared by Student t test **(G)** or one-way Anova and Tukey post-test **(H)** *p<0.5, **p<0.01, ***p<0.001, ****p< 0.0001). Horizontal bars indicate the means.

Female C57 BL/6 mice that were intravesically instilled with PBS or 3x10^8^ CFU of Ty21a^FR^ were sacrificed 72h later and their bladder analyzed by immunohistochemistry, as well as for hemoglobulin content. As expected from our previous experiments showing increased immune cell infiltration upon Ty21a^LYO^ (12), and despite high variability, a significant (ca. 10-fold) increase of the number of CD8^+^ T cells upon Ty21a^FR^ was observed (n= 3 mice) (**Figures 1A, B**). However, the number of blood vessels (CD31^+^, **Figure 1C, D**) in the same bladder, as well as the total blood content (as measured by the hemoglobin content, **Figure 1E**) were not increased by Ty21a^FR^. Moreover, no HEV (Pnad^+^) were observed in the bladder with or without Ty21a^FR^ treatment (**Figure 1F**, right panel), while they were detected in LNs (**Figure 1F**, left panel). These results suggest that the rapid increase of CD8^+^ T cells upon Ty21a instillation is not associated with neoangiogenesis.

We then examined whether vessel permeability was altered using an Evans Blue assay. The data showed that vessel permeability was significantly increased in the bladder one day after Ty21a^FR^ instillation, but not later (**Figure 1G**). Similarly, elevated vessel permeability was observed with Ty21a^LYO^ treatment while heat-killed bacteria had no effect (**Figure 1H**). No increased permeability was observed at distant organs, such as the lung or the liver (**Supplementary Figures 1A, B**) and thus the increased vascular permeability was limited to the instillation site. Vessel permeability in the bladder was similarly increased by Ty21a^FR^ and Ty21a^LYO^ which may in part explain the increased T-cell infiltration observed in independent experiments with either of the two formulations (11, 12, 19). To address this hypothesis, we therefore directly compared the ability of Ty21a^FR^ and Ty21a^LYO^ to increase immune cell infiltration in the bladder.

### 3.2 Bladder-immune cell infiltration and survival of bladder tumor-bearing-mice are increased by Ty21a^FR^ compared to Ty21a^LYO^ treatment

**Figure 2:**
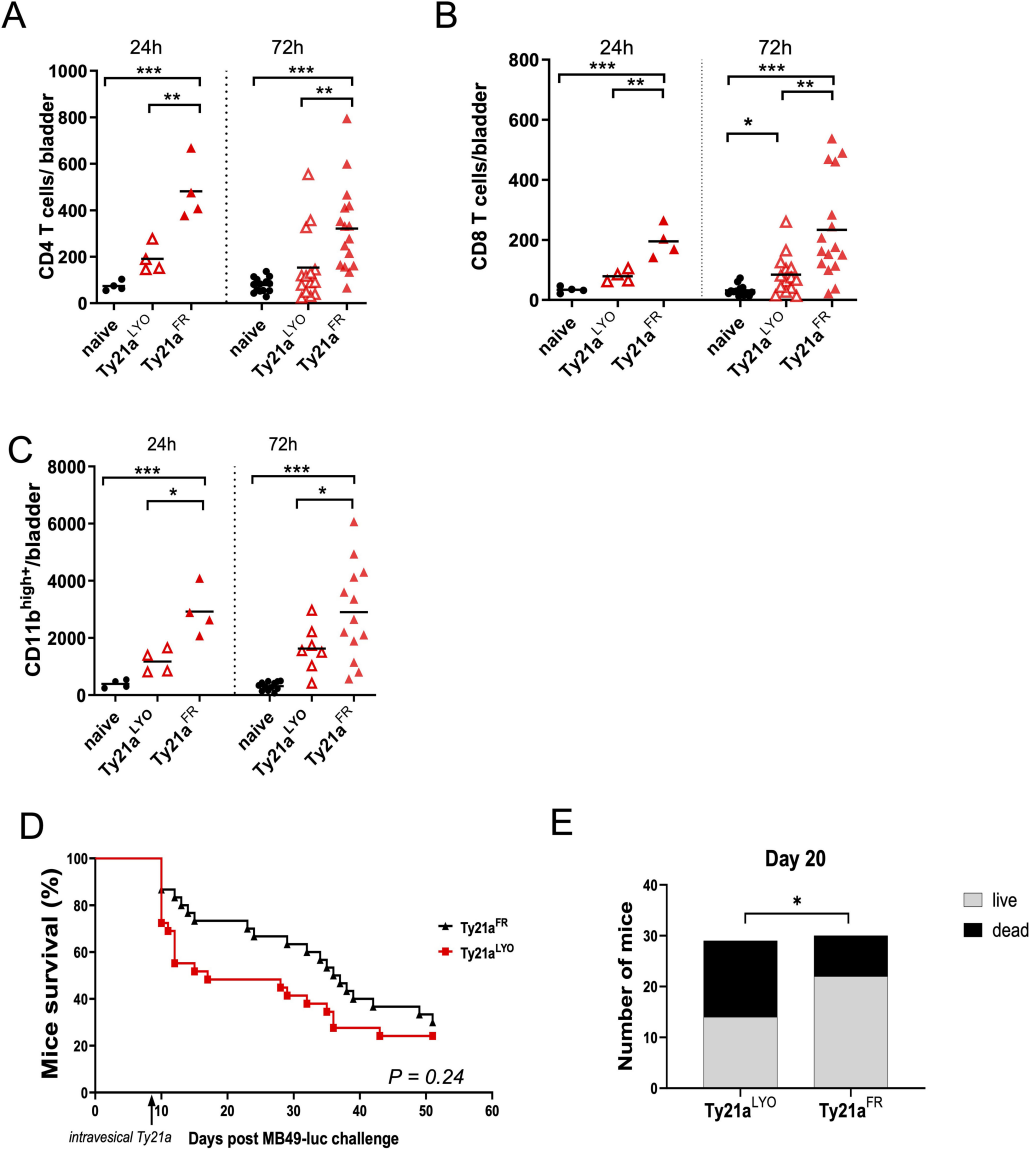
Bladder-immune cell infiltration and survival of bladder-tumor bearing mice upon Ty21a instillation. Immune cell infiltration of CD4^+^ T cells **(A)**, CD8^+^ T cells **(B)** or myeloid CD11b^high^ cells **(C)** in the bladder was examined in naïve mice or mice that had received, 24h (left panels, n=4 mice/group) or 72h (right panels, n=7–16 mice/group) before, a single intravesical instillation of $3\times 10^{8}$ CFU of Ty21a^FR^ orTy21a^LYO^. Numbers of indicated cells/bladder in individual mice are shown. Groups were compared by one-way Anova and Tukey post-test. *p<0.5, **p<0.01, ***p< 0.001. Horizontal bars indicate the means. **(D)** Survival of MB49 bladder tumor bearing mice is shown upon a single intravesical instillation of $3\times 10^{8}$ CFU of Ty21a^FR^ (n= 30 mice) or Ty21a^LYO^ (n = 29 mice) 8 days after tumor instillation. Comparison by a Chi-square test at day 20 is shown **(E)**.

Immune cell infiltration in the bladder of mice was examined 24h and 72h after intravesical instillation of 3x10^8^ CFU of Ty21a^FR^ or Ty21a^LYO^, or in untreated naïve mice. The data showed that CD4^+^ (**Figure 2A**) and CD8^+^ T cells (**Figure 2B**), as well as myeloid cells (CD11b^high^, **Figure 2C**), were all significantly increased (ca. 2-fold) by Ty21a^FR^ as compared to Ty21a^LYO^, at both time points, despite considerable interindividual variability at 72 hours. Overall, Ty21a^FR^ instillation induced a significantly augmented (5-10-fold) immune cell infiltration compared with naïve controls, consistent across all cell types and time points. More importantly, in the presence of bladder tumor, Ty21a^FR^ was significantly more efficient than Ty21a^LYO^ at improving short-term survival of mice bearing large orthotopic bladder tumors, while at long-term only a trend was observed (**Figures 2D, E**). Whether higher immune cell infiltration induced by Ty21a^FR^ at 72 hours correlates with the improved short-term survival of mice warrants further investigation. In naïve mice, a combinatory treatment setting involving subcutaneous vaccination with the Uty antigen followed by intravesical instillation of either formulation, Ty21a^FR^ was superior to Ty21a^LYO^ in enhancing the numbers of total CD4^+^ and CD8^+^ T cells, as well as vaccine-specific CD8^+^ T cells (DexUty^+^) and IFN-γ–secreting CD8^+^ T cells in the bladder (**Supplementary Figure 2**). Altogether, the data show that despite similar increased vessel permeability, the two formulations differed in the magnitude by which they increase immune cell infiltration. We thus further examined whether differences in chemokine secretion in the bladder could underlie this discrepancy.

### 3.3 C5a, CXCL5 and CXCL2 chemokines and myeloid cells expressing their receptors are significantly increased by Ty21a^FR^ as compared to Ty21a^LYO^

**Figure 3:**
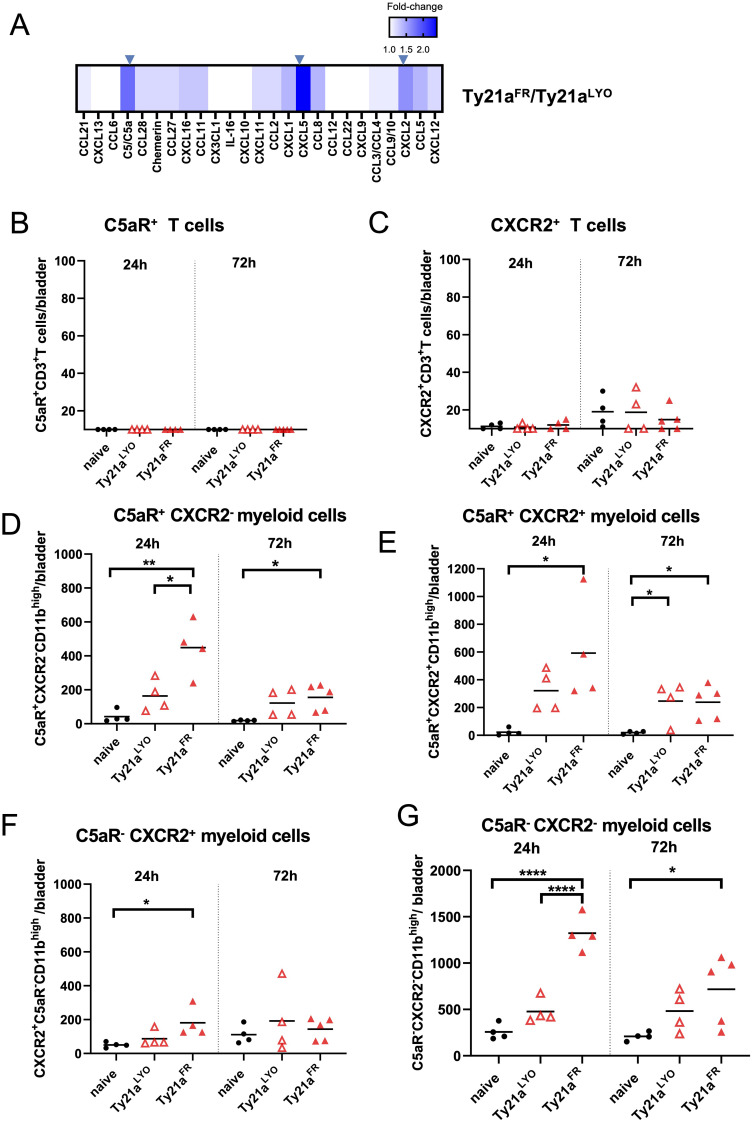
Chemokines and receptor-specific immune cell infiltration in the bladder upon Ty21a instillation. **(A)** Heatmap of the relative changes of the indicated chemokines levels in the bladder between Ty21a^FR^ and Ty21a^LYO^ (24h after a single instillation, n=3 mice/group) is shown. Significantly increased (≥1.5 fold) chemokines are indicated by an arrow**. (B–G)** Bladder of individual naïve mice or mice that had received, 24h (left panels, n=4 mice/group) or 72h (right panels, n=4–5 mice/group) before, a single intravesical instillation of $3\times 10^{8}$ CFU of Ty21a^FR^ or Ty21a^LYO^ were examined for single and double expression of C5aR and CXCR2 expression in T cells **(B, C)** or CD11b^high^ myeloid cells **(D–G)**. Groups were compared by one-way Anova and Tukey post-test. *p<0.5, **p<0.01, ****p< 0.0001. Horizontal bars indicate the means.

The levels of 25 chemokines were analyzed in the bladders of mice 24h after intravesical instillation of 3x10^8^ CFU of Ty21a^FR^ or Ty21a^LYO^ using a chemokine array. Relative changes of these chemokines between the two formulations (**Figure 3A**) showed that C5a, CXCL5 and CXCL2 were significantly increased (> 1.5-fold) in bladders treated with Ty21a^FR^ compared to those treated with Ty21a^LYO^. Their cognate receptors (C5aR for C5a and CXCR2 for both CXCL5 and CXCL2), however, were either undetectable or lowly expressed on T cells from naïve mice and were not modulated by either Ty21a^FR^ or Ty21a^LYO^ (**Figures 3B, C**), precluding their direct effect on T-cell infiltration. In contrast, C5aR^+^CD11b^high^ myeloid cells were significantly more numerous 24h after instillation in bladders treated with Ty21a^FR^ as compared to Ty21a^LYO^ (**Figures 3D, E**), while CXCR2^+^CD11b^high^ cells were less impacted (**Figure 3F**). In addition, Ty21a^FR^ significantly increased C5aR^-^CXCR2^-^CD11b^high^ myeloid cells (**Figure 3G**) as compared to Ty21a^LYO^, suggesting that other analytes not present in our array may be involved and, that different types of myeloid cells may indirectly participate in T-cell attraction.

### 3.4 Immune cell infiltration upon Ty21a^FR^ is significantly decreased in TLR4- and/or MYD88-KO mice

**Figure 4:**
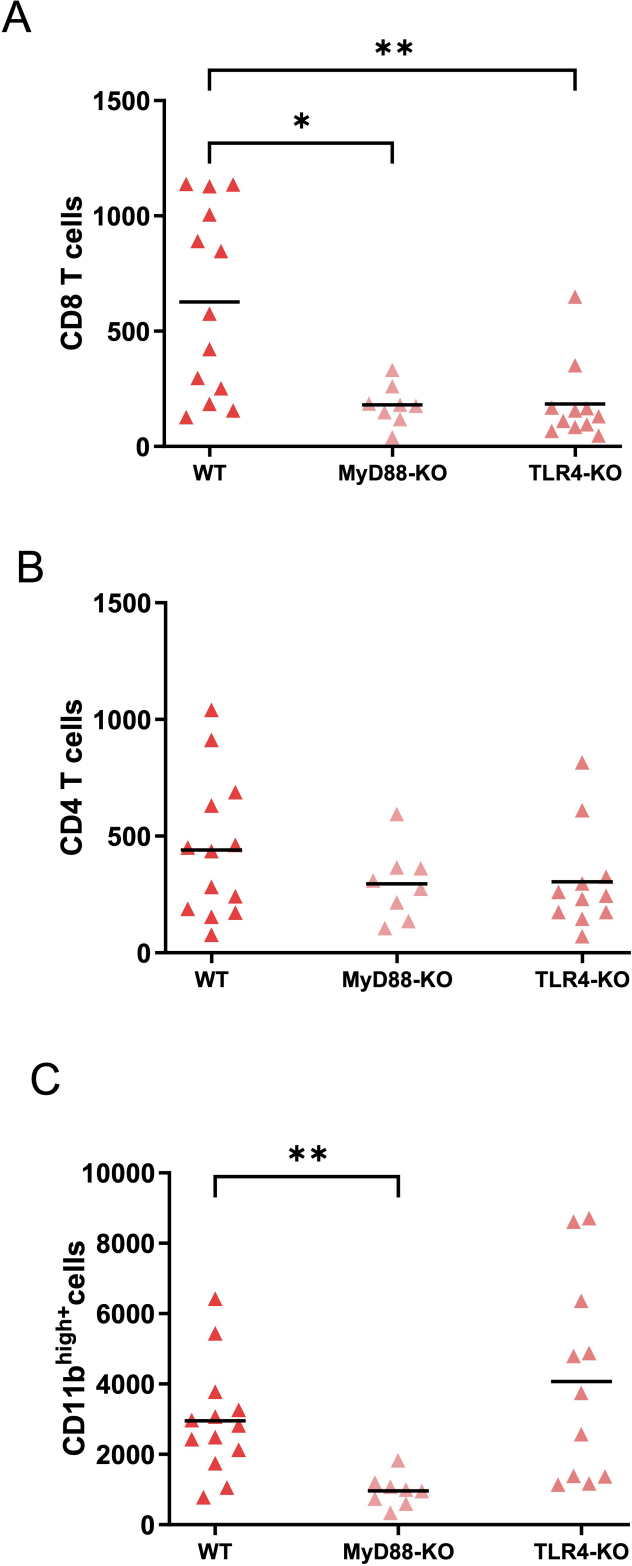
Immune cell infiltration in the bladder of wild type (WT), MyD88- and TLR4-KO mice upon Ty21a^FR^. Immune cell infiltration in the bladder (without tumor) of WT (n=13), MyD88 (n=8) and TLR4-KO (n=11) mice was examined 72h after a single intravesical instillation of 3x10^8^ CFU of Ty21a^FR^. Numbers of CD8^+^ T cells **(A)**, CD4^+^ T cells **(B)** and myeloid CD11b^high+^ cells **(C)** per bladder in individual mice are shown. Groups were compared by Kruskal-Wallis test and Dunn’s post-test. *p<0.5, **p<0.01. Horizontal bars indicate the means.

In the early oral typhoid vaccine field trials, freshly harvested Ty21a was more effective than enteric capsules containing lyophilized Ty21a, which may be related to the expression of a more active form of LPS in the fresh Ty21a (30, 31, 41–43). To examine whether TLR4-dependent LPS sensing was involved in the infiltration of immune cells in the bladder, we compared the effects of intravesical Ty21a^FR^ in wild-type (WT), TLR4-KO and MyD88-KO mice. MyD88-KO mice were included to evaluate the role of MyD88, an essential adaptor for signaling through all TLRs (except TLR3) and IL-1 receptors. CD8^+^ T cells were significantly decreased upon Ty21a^FR^ in both TRL4- and MyD88-KO mice compared to WT (**Figure 4A**) while the levels of CD4^+^ T cells upon Ty21a^FR^ were also decreased in TLR4- and MyD88-KO mice, though not significantly (**Figure 4B**). In contrast, the levels of CD11b^high^ myeloid cells were significantly reduced only in MyD88-KO mice. These data suggest that T-cell infiltration induced by Ty21a is at least partially mediated by LPS, while myeloid cell infiltration occurs by TLR4-independent but MyD88-dependent mechanisms.

## 4 Discussion

The use of intravesical Salmonella vaccines for bladder cancer treatment has now reached Phase I trials not only with Ty21a (NCT03421236) (13, 14), but also with another attenuated S. Typhi strain, ZH9 (a proprietary bacterial immunotherapy of Prokarium Ltd, NCT06181266). Here, we investigated some of the mechanisms underlying Ty21a immunostimulatory effects in the bladder, using the MB49 orthotopic murine model (32), which closely reproduces non-muscle invasive bladder tumors of patients and that may be informative for future therapeutic strategies (44). Our focus was on understanding how intravesical instillation of Ty21a in bladder promotes immune cell infiltration, particularly T cells, and how it may depend on the formulation of the bacteria used. Our data show that both Ty21a^FR^ and Ty21a^LYO^, but not heat-killed bacteria, transiently increased local vascular permeability. The disruption of the vascular endothelial barrier can permit the influx of immune cells (45) and participates in the immunostimulatory effects of Ty21a. This agrees with previous reports of histologically scored inflammation of the bladder (presence of edema, fibrosis and T- and myeloid-cell infiltration) upon Ty21a^LYO^ instillations (12).

Direct comparison of the two formulations administered after Uty vaccination showed that Ty21a^FR^ increased total and Uty-specific CD8^+^ T cells by ~10-fold, similarly to previous data obtained from treatment with prototype E7 vaccine (19). Ty21a^LYO^ was significantly less efficient, recruiting half the number of CD8^+^ T cells to the bladder when compared to Ty21a^FR^. Intravesical Ty21a used as monotherapy in the bladder, demonstrated increased T- and myeloid cell infiltration locally by Ty21a^FR^ within 24h of instillation (5-10-fold), as compared to Ty21a^LYO^ (ca. 2-fold). Despite this, previous studies using the MB49 murine bladder tumor model, have shown that Ty21a^LYO^ was sufficient to result in ca. 80% survival when administered one day after tumor-challenge (12), while the treatment of established day-5 bladder tumors was more efficient than the standard BCG therapy (11). Using a more stringent tumor context of day-8 established tumors, we report that intravesical Ty21a^FR^ significantly prolonged mice survival as compared to Ty21a^LYO^, suggesting that the formulation of Ty21a may be critical for a increased efficacy in various settings.

T cells are critical players of intravesical treatment with Ty21a, whether used alone (12) or in combination with vaccination (19). We hypothesized that their higher recruitment by Ty21a^FR^ compared to Ty21a^LYO^, might be associated with differential chemokine induction. Indeed, our data showed that C5a, CXCL5 and CXCL2a were more strongly increased by Ty21a^FR^ than by Ty21a^LYO^. The same chemokines, which are known chemoattractants of neutrophils (46, 47), were previously indicated in the comparison of intravesical Ty21a^FR^ to intravesical CpG (a synthetic TLR9 agonist) (19). These chemokines may not, however, directly recruit T cells as their receptors (C5aR and CXCR2) were minimally expressed on T cells. In contrast, our data revealed greater infiltration of myeloid cells expressing both receptors following Ty21a^FR^ treatment compared with Ty21a^LYO^, as well as increased numbers of double-negative myeloid cells. Notably, bladder-infiltrating myeloid cells including neutrophils, macrophages and dendritic cells (12) were already elevated 24h-post Ty21a^LYO^ treatment. This myeloid infiltration may contribute to T-cell attraction, as neutrophil-mediated T-cell recruitment to the bladder has been previously reported upon intravesical BCG administration (48). Whether a similar mechanism is involved in the response to Ty21a remains to be elucidated. The observed increase in C5a, CXCL5 and CXCL2a as well as their receptors on CD11b^high^ myeloid cells, suggests that these cells may contribute indirectly to T-cell recruitment by amplifying local inflammation or by producing additional mediators not captured in our chemokine array. Further studies are needed to clarify the relationship between myeloid cell activation and T-cell infiltration in the context of intravesical Ty21a therapy and to identify the specific chemokines involved.

The lyophilization or freeze-drying process may affect lipopolysaccharides of gram-negative bacteria such as Ty21a (43, 49, 50), potentially reducing the TLR4 agonistic activity of lipid A, which may explain a lower efficacy of Ty21a^LYO^. In line with this assumption, CD8^+^ T-cell infiltration was significantly reduced in both TLR4- and MyD88-KO mice, following Ty21a^FR^ instillation, while infiltration of myeloid cells was unaffected in TLR4-KO, but abrogated in MyD88-KO mice. These findings strongly suggest that T-cell recruitment is at least partially mediated via LPS-TLR4 signaling, whereas myeloid cell infiltration depends on MyD88 but involves TLR4-independent pathways. This is consistent with the ability of *Salmonella* to activate additional pattern-recognition receptors, including TLR5 through flagellin (51), TRL9 via bacterial DNA (52) and likely TLR1/2 through lipopeptides/lipoproteins (53). Further studies on both formulations are needed to delineate the roles of these pathways in intravesical Ty21a-induced myeloid cell recruitment.

Freshly prepared Ty21a inoculum to prevent typhoid fever was previously reported in oral vaccination trials against typhoid fever and found to be more effective (30, 31), when compared to a lyophilized encapsulated bacteria formulation (Vivotif^®^), which was eventually chosen and developed for mass oral vaccination. This was clearly logistically necessary for an oral prophylactic vaccine to be administered to millions of people; however, the context may be different for a cancer immunotherapy delivered through intravesical instillations, that may benefit from alternative formulations. Nevertheless, applying such approaches in clinical trials may be particularly challenging, particularly regarding reliable live preparation of bacterial inoculum, determining their viability, and the potential assessment of LPS integrity prior to administration.

In the context of BCa, studies on BCG formulation and therapeutic efficacy are scarce. It has been reported in subcutaneous bladder tumor mouse model that fresh and lyophilized BCG have similar tumor growth-inhibiting effects, when co-administrated with the tumor cell line (54). *In vitro*, both formulations can attach strongly to MBT-2 mouse bladder tumor cell line, although fresh BCG attachment occurred earlier (54). Further studies are thus needed to clarify which BCG formulation may provide the best therapeutic effect upon intravesical instillation.

Overall, our study emphasizes the importance of bacterial formulation in the intravesical immunotherapy setting. We demonstrate that while both formulations of Ty21a increase vascular permeability similarly, only Ty21a^FR^ promotes stronger immune cell recruitment and superior tumor regression. One limitation is the variable content of excipients in Ty21a^LYO^, which are unlikely to be present in the Ty21a^FR^ formulation. Some of these excipients have been reported to exert minor immunomodulatory effects at high doses *in vitro* or *in vivo* when administered orally or systemically (55–59). However, none have been evaluated for intravesical administration. Given that a tenfold dilution of the capsule content was administered into the mouse bladder, it is unlikely that these excipients exerted any significant immunomodulatory effect. These findings underscore the potential for a more rational design and optimization of live bacterial therapies for bladder cancer, including both novel agents such as Ty21a and refining gold-standard BCG-based strategies, even in the face of logistical challenges.

## Data Availability

The raw data supporting the conclusions of this article will be made available by the authors, without undue reservation.
