# Supplementary material for: Characterization of Ty21a immunostimulatory effects in the mouse bladder
Source: Front Immunol. 2025 Nov 26;16:1629462. doi: 10.3389/fimmu.2025.1629462 (PMC12689529; doi:10.3389/fimmu.2025.1629462)
Supplement: Supplementary file 1 [file DataSheet1.pdf]

Figure 1 sup

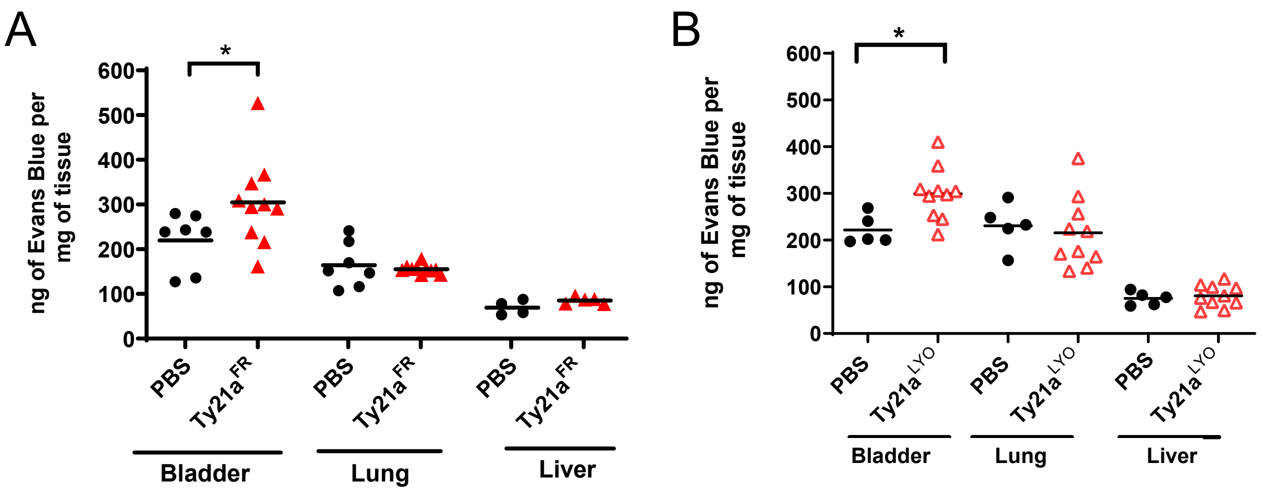

Supplementary Figure 1: Vessel permeability upon Ty21a

To assess vessel permeability, Evans Blue assay was performed 1 day after intravesical instillations of PBS, $3x10^8$ CFU of $Ty21a^{FR}$ (A) or $Ty21a^{LYO}$ (B). Evans Blue recovery (µg per mg of tissue) in the indicated organs is shown. Groups were compared by a Student t test. *p<0.05,

Figure 2 sup

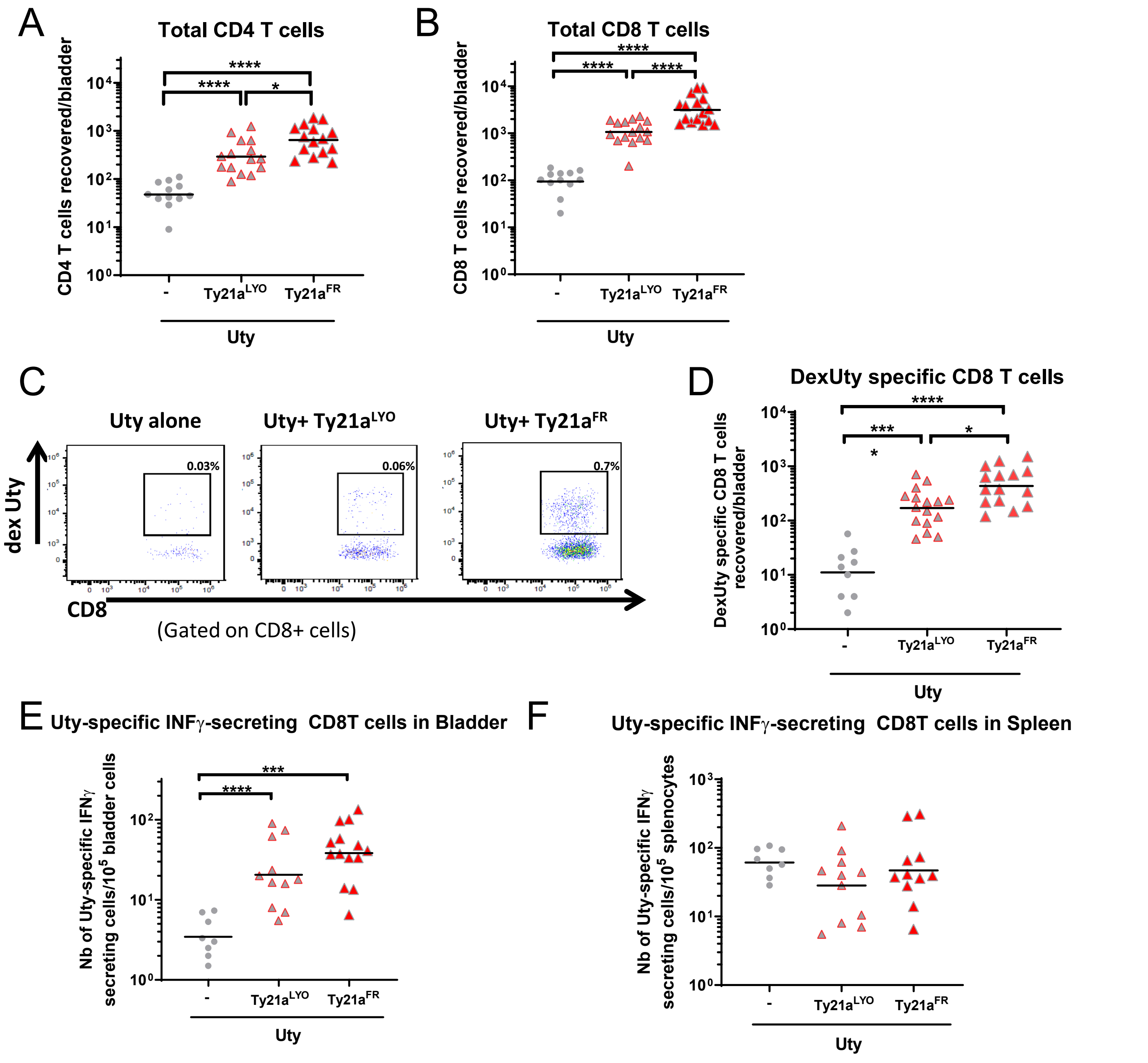

Supplementary Figure 2: Immune cell infiltration in vaccinated mice upon intravesical Ty21a. Mice s.c. immunized with the adjuvanted Uty vaccine (50µg $Uty_{246-254}$ + 10µg CpG + 0.4 µg HLT), received 5 days later intravesical $Ty21a^{LYO}$ or $Ty21a^{FR}$ or remained untreated (-) and were then sacrificed 3 days later. Numbers of total $CD4^+$ (**A**) and $CD8^+$ (**B**) T cells recovered in the bladder are shown. Numbers of vaccine (Uty)-specific CD8 T cells were determined with DexUty staining (representative flow cytometry plots in **C**) and quantified (**D**). Numbers of functional Uty-specific $CD8^+$ T cells are shown by ELISPOT in the bladder (**E**) and in the spleen (**F**). Groups were compared by one-way Anova and Tukey post-test after log_{10} transformation. *p<0.5, *** p< 0.001, **** p< 0.0001.
